# Fetal Neuronal Vesicles in the Assessment of Perinatal Brain Dysfunction and Late-Onset Growth Restriction: A Pilot Study

**DOI:** 10.3390/ijms27020679

**Published:** 2026-01-09

**Authors:** Vladislava Gusar, Natalia Kan, Anastasia Leonova, Vitaliy Chagovets, Victor Tyutyunnik, Anna Zolotareva, Nataliya Tyutyunnik, Ekaterina Yarotskaya, Gennadiy Sukhikh

**Affiliations:** 1 Laboratory of Applied Transcriptomics, Federal State Budget Institution “National Medical Research Center for Obstetrics, Gynecology and Perinatology Named after Academician V.I. Kulakov” of the Ministry of Health of the Russian Federation, 4, Oparina Street, Moscow 117997, Russia; 2 Federal State Budget Institution “National Medical Research Center for Obstetrics, Gynecology and Perinatology Named after Academician V.I. Kulakov” of the Ministry of Health of the Russian Federation, 4, Oparina Street, Moscow 117997, Russia; n_kan@oparina4.ru (N.K.); serebriakovanna@gmail.com (A.Z.); inter_otdel@mail.ru (E.Y.); g_sukhikh@oparina4.ru (G.S.); 3 Department of Molecular Diagnostic Methods and Personalized Medicine, Federal State Budget Institution “National Medical Research Center for Obstetrics, Gynecology and Perinatology Named after Academician V.I. Kulakov” of the Ministry of Health of the Russian Federation, 4, Oparina Street, Moscow 117997, Russia; aa_leonova@oparina4.ru (A.L.); tysia07@bk.ru (N.T.); 4 Laboratory of Metabolomics and Bioinformatics, Federal State Budget Institution “National Medical Research Center for Obstetrics, Gynecology and Perinatology Named after Academician V.I. Kulakov” of the Ministry of Health of the Russian Federation, 4, Oparina Street, Moscow 117997, Russia; v_chagovets@oparina4.ru; 5 Center for Scientific and Clinical Research, Federal State Budget Institution “National Medical Research Center for Obstetrics, Gynecology and Perinatology Named after Academician V.I. Kulakov” of the Ministry of Health of the Russian Federation, 4, Oparina Street, Moscow 117997, Russia; tioutiounnik@mail.ru

**Keywords:** fetal neuronal vesicles, sumoylation, neddylation, placental dysfunction, fetal growth restriction

## Abstract

Fetal growth restriction (FGR) remains a significant problem in obstetrics and is a key risk factor for perinatal brain injury. The fetal neuronal vesicles (FNVs) isolated from maternal blood represent an innovative approach—a “fetal brain liquid biopsy”—enabling early diagnostics of neuronal dysfunction in FGR. Western blotting was used to evaluate the protein pattern expression of FNVs isolated from the blood of pregnant women with FGR and uncomplicated pregnancy. Significant changes in the neurotrophic proteins levels (pro-BDNF, pro-NGF) and presynaptic neurotransmission proteins (SYN1, SYP, SYNPO) were identified. New data were obtained on changes in the expression of proteins of sumoylation (SUMO2/3/4) and neddylation (NAE1, UBC12), which differs in early-onset and late-onset FGR. Moreover, increased SUMO2/3/4 levels can be considered as an endogenous neuroprotective response to cerebral hemodynamic reaction in fetuses with late-onset growth restriction. An association has been established between changes in the expression of the studied proteins and intraventricular hemorrhage (IVH) in newborns with late-onset growth restriction.

## 1. Introduction

Despite significant advances in medical technologies, including the development of prenatal diagnostics, fetal growth restriction (FGR) remains one of the most significant problems in current obstetrics. This condition is a key risk factor for stillbirth, high perinatal morbidity, and neurological disorders in the later stages of the child’s development [1,2,3].

According to the current classification, early-onset (before 32 weeks) and late-onset (after 32 weeks of pregnancy) forms of FGR are distinguished. These two forms differ in clinical and pathogenetic features, which determine neonatal and postneonatal complications [4]. Though early-onset FGR is associated with long-term placental insufficiency, a statistically significant predominance of delayed physical development [5], psychomotor and cognitive impairments [6,7,8] are observed in children with late-onset FGR [9]. This can be explained by the gradual development of the fetal nervous system. Cortical folding reaches its peak in the third trimester, laying the ground for the architecture of neural networks and subsequent neurological function in normal brain development [10,11]. Disorders during this period, caused by placental insufficiency accompanying late-onset FGR, damage grey and white brain matter, leading to severe neurologic conditions [10].

Diagnosing late-onset FGR is difficult because, due to the absence of significant alterations in ultrasound dopplerography, this condition is often interpreted as a constitutionally low birth weight fetus [12]. At the same time, routine assessment of blood flow dynamics in the anterior cerebral artery, which is the first to respond to reperfusion processes, preceding the changes in the middle cerebral artery, is technically complex and limits diagnostic possibilities. The cerebrovascular hemodynamic response, a so-called “brain-sparing effect,” presumed to protect the brain by increasing oxygen levels due to the redistribution of cardiac output, can, on the contrary, aggravate neuronal damage through oxidative stress and reperfusion mechanisms [13,14,15]. Thus, in pregnancies complicated by late-onset FGR, some compensatory effects and prolonged detection of Doppler signs of cerebral hemodynamic impairment mask the starting process of brain tissue damage [16,17,18].

It is important to note that the mechanisms of perinatal fetal brain injury along the placenta–fetus–brain axis are mediated by extracellular vesicles of neuronal origin [10]. Their biogenesis depends on the cell’s state, and their release with a specific cargo of proteins, lipids, and nucleic acids is strictly specific [19]. During the damage to fetal brain tissue, vesicles with an altered cargo are secreted through the blood–brain barrier [20]. Investigation of this cargo will provide insight into the degree of brain dysfunction based on assessing the levels of marker molecules and make use of their diagnostic and prognostic potential.

Pioneering research by Goetzl L. et al. on non-invasive prenatal monitoring of fetal neurodevelopmental disorders demonstrated changes in protein expression in fetal neuronal vesicles originating from maternal plasma in fetal alcohol syndrome [21,22]. Our previous work was the first to demonstrate the feasibility of non-invasive assessment of the levels of proteins critical for neuronal survival, function, and synaptic plasticity in fetal neuronal vesicles isolated from maternal plasma at early-onset FGR. A relationship was established between their altered levels and parameters of neonatal weight, length, and complications (cerebral ischemia, intraventricular hemorrhage (IVH), asphyxia, and CNS depression syndrome) in children with early-onset growth restriction. Furthermore, we obtained new data on significant changes in the expression of proteins that perform posttranslational modifications, sumoylation and neddylation, crucial for brain development, synaptic protein stability, and neurotransmission [23].

The idea of the involvement of neurotrophins (BDNF, NGF, NT-3, NT-4), proteins of pre- and postsynaptic neurotransmission (SYN1, SYT1, SYP, SYNPO, PSD95, NPTX2), and key components of post-translational modifications, including sumoylation (SUMO1, SUMO2/3/4, UBC9) and neddylation (NEDD8, NAE1, UBC12) proteins in the implementation of mechanisms of perinatal brain damage in fetuses with late-onset growth restriction is interesting in the context of pathogenetic differences between early-onset and late-onset FGR, and the significant changes in the levels of fetal neuronal proteins, which we have previously discovered in early-onset FGR. In this regard, the aim of our study was to identify changes in the expression patterns of fetal proteins in neuronal vesicles, assess their expression depending on the presence of intraventricular hemorrhage in newborns with late-onset FGR, and perform a comparative analysis of the studied protein levels in early-onset and late-onset FGR. It is important to emphasize that the area of neuronal vesicle research mainly refers to neurodegenerative diseases and, therefore, our study in late-onset FGR is pioneering.

## 2. Results

### 2.1. Ultrasound and Doppler Data of the Studied Patients

Taking into account the pathogenesis of early-onset and late-onset FGR, influenced by the gestational stages of fetal development and associated differences in cerebral hemodynamics, the assessment of ultrasound and Doppler data was carried out in pregnant women with late-onset FGR and a comparison group (Table 1).

At similar gestational age (36.6 weeks in both groups) and at the analogues mothers age (32 and 35 years, respectively), significant changes in Doppler parameters were found in the group with late-onset FGR: an increase in the pulsatility index of the umbilical artery (PI UA) (1.33; 0.79; *p* < 0.001), and a decrease in the cerebroplacental ratio (CPR) (1.0; 1.9; *p* < 0.001). At the same time, the values of the pulsatility index of the middle cerebral artery (PI MCA), which is routinely used to assess the hemodynamics of the fetus with late-onset growth restriction, were statistically insignificant (*p* = 0.368). It is important to note that fetoplacental blood flow disorders were observed in all pregnant women with late-onset FGR (<0.001). Despite the absence of statistically significant differences in the main fetometric parameters (head circumference, abdominal circumference, and biparietal diameter), the estimated weight of the fetus with growth restriction showed a tendency to decrease (2.07; 2.61; *p* = 0.074).

### 2.2. Neonatal Outcomes

The average birth weight of FGR newborns was 2.11 kg, compared to 2.72 kg in the comparison group (*p* < 0.001). All newborns with growth restriction met the criteria for very low birth weight (*p* < 0.001). A neuro-sonographic evaluation of the newborns with FGR was performed, and their neurological status was assessed. The clinical characteristics of the newborns and neonatal outcomes are presented in Table 2.

Of particular interest were subependymal cysts (62.5%; *p* = 0.02) and IVH (62.5%; *p* = 0.02) in newborns with late-onset FGR, which were absent in the comparison group.

### 2.3. String Database

**Figure 1:**
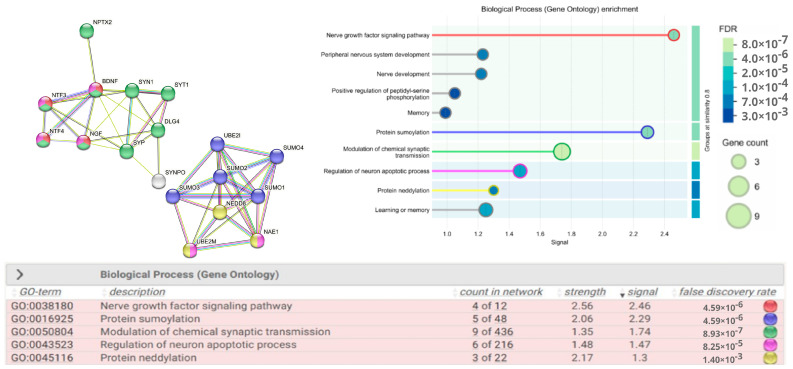
Protein–protein interaction network. Network evidence—STRING false discovery rate (FDR) < 0.01. Protein identifiers are given according to UniProtKB/Swiss-Prot. BDNF—Brain-Derived Neurotrophic Factor (P23560), NGF—Nerve Growth Factor (P01138), neurotrophins NTF-3 (P20783) and NTF-4 (P34130), SYN1—Synapsin 1 (P17600), SYT1—Synaptotagmin 1 (P21579), SYP—Synaptophysin (P08247) and SYNPO—Synaptopodin (P08247), PSD95 (DLG4)—Post-Synaptic Density Protein (P78352), NPTX2—Neuronal Pentraxin (P47972), SUMO 1-4—Small Ubiquitin Like Modifier (P63165; P61956; P55854; Q6EEV6), UBC 9 (UBE2I)—Ubiquitin Conjugating Enzyme E2I (P63279), NEDD 8—Neural Precursor Cell Expressed, Developmentally Down-Regulated 8 (Q15843), NAE1 (APPBP1)—NEDD8 Activating Enzyme E1 Subunit 1 (Q13564), UBC 12 (UBE2M)—Ubiquitin Conjugating Enzyme E2M (P61081). The color scheme of the circles in the table corresponds to the names of the biological processes in which the proteins in the network diagram participate. The signal is defined as a weighted harmonic mean between the observed/expected ratio and −log(FDR).

Taking into account the stages of fetal nervous system development, key proteins involved in neurogenesis (BDNF, NGF, NT-3 and NT-4), pre-/postsynaptic neurotransmission (SYT1, SYN1, SYP, SYNPO, PSD95 (DLG4)), NPTX2), and those performing post-translational modifications, sumoylation (SUMO 1, SUMO 2/3/4, UBC9) and neddylation (NEDD8, NAE1 (APPBP1), UBC12) were selected in accordance with the STRING v.12 protein–protein interaction database for subsequent assessment of their expression in the samples of FNVs isolated from maternal blood plasma in late-onset FGR (Figure 1).

Our attention was focused on the study of a number of key proteins that promote the survival and differentiation of neurons, axon growth, and regulation of synaptic transmission and plasticity (BDNF, NGF, NT-3, NT-4), those involved in synaptogenesis, the neurotransmitter release cycle (SYT1, SYN1, SYP), and synaptic plasticity (PSD95 (DLG4), and those that participate in the regulation of short-term and long-term synaptic plasticity (SYNPO) and in the formation of excitatory synapses and clustering of AMPA-type glutamate receptors in formed synapses (NPTX2). Another scope of the study comprised the proteins that post-translational modifications such as sumoylation ((SUMO 1-4, UBC9 (UBE2I), playing a critical role in various cellular processes such as nuclear transport, transcription regulation, DNA repair, apoptosis, protein stability) and neddylation ((NEDD 8, NAE 1 (APPBP1), UBC 12 (UBE2M), involved in the control of the cell cycle and embryogenesis, regulation of growth, viability and development of cells). In addition, both post-translational modifications are critical for neuronal morphology, spinogenesis, and stability of pre- and postsynaptic proteins regulating synaptic transmission and plasticity under physiological and pathological conditions [24,25].

### 2.4. Proteins Expression in FNVs

**Figure 2:**
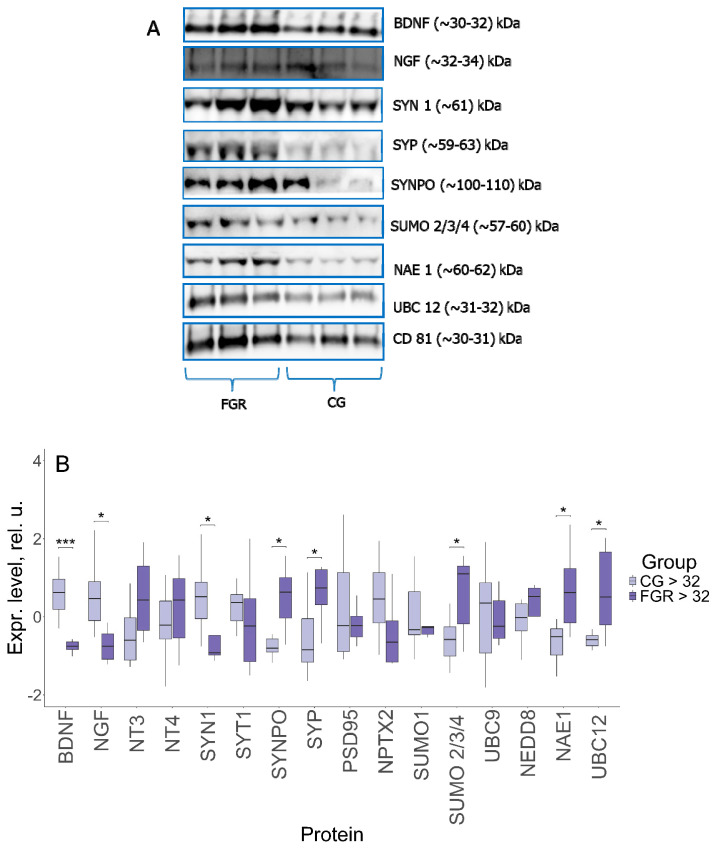
(**A**) Western blot of the membrane. The membranes represent a set of three samples from each group. (**B**) Comparative analysis of proteins in FNVs from pregnant women with late-onset FGR (*n* = 8) and comparison group (CG) (*n* = 8). Data are presented in the format Me (Q1, Q3); *: significance level *p* ≤ 0.05, ***: significance level *p* ≤ 0.001, when compared with CG.

Evaluation of the protein expression pattern was conducted by Western blotting in the FNVs, isolated from the blood plasma of pregnant women with late-onset FGR and of the comparison group (Figure 2).

Comparative analysis revealed a significant decrease in the expression of key neurotrophins—BDNF (−0.75, 0.62; *p* ≤ 0.001) and NGF (−0.76, 0.46; *p* ≤ 0.01) in late-onset FGR versus the comparison group. In the group of proteins involved in the modulation of synaptic transmission and neurotransmitter release, statistical differences were found for SYN1 (−0.92, 0.52; *p* ≤ 0.02): its expression was decreased in late-onset FGR compared to uncomplicated pregnancy, and SYP (0.74, −0.85; *p* ≤ 0.01), the expression of which, on the contrary, was increased. In late-onset FGR, the level of SYNPO, which is involved in synaptic plasticity, was also increased (0.63, −0.8; *p* ≤ 0.03). It is interesting to note that expression of NPTX2, involved in the formation of excitatory synapses and clustering of glutamate receptors, almost reached the borderline statistical significance (−0.65, 0.45; *p* ≤ 0.08). In the group of sumoylation proteins, only the expression of SUMO 2/3/4 (1.11, −0.58; *p* ≤ 0.02) was significantly higher in late-onset FGR than in uncomplicated pregnancy. At the same time, the activating NAE 1 and conjugating UBC 12 neddylation enzymes were also significantly increased in FGR: 0.62, −0.51; *p* ≤ 0.01, and 0.5, −0.58; *p* ≤ 0.01, respectively.

It is important to emphasize that the fragments corresponding in size to the conjugated forms were detected for a number of proteins, in particular, SYP (~59–63 kDa), SUMO 2/3/4 (~55–60 kDa), UBC9 (~30–32 kDa) and UBC12 (~31–32 kDa). The sizes of BDNF and NGF fragments were ~30–32 and ~32–34 kDa, respectively. Such molecular weights are characteristic not of mature forms, but of precursors—pro-BDNF and pro-NGF. All samples were positive for CD81.

### 2.5. Comparative Analysis of FNV Protein Expression in Early- and Late-Onset FGR

**Figure 3:**
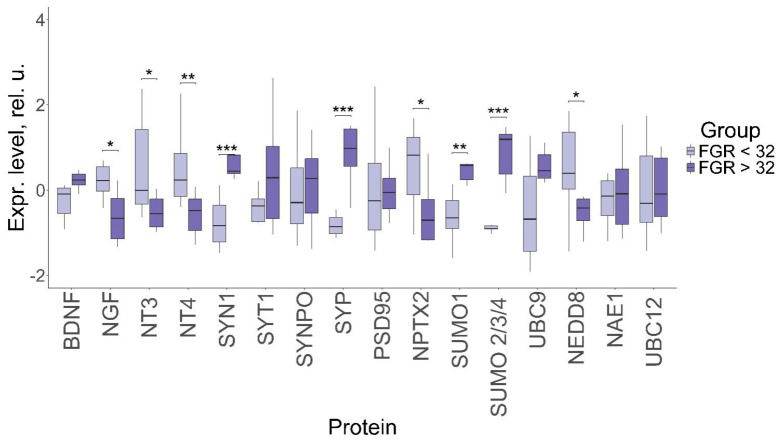
Comparative analysis of protein levels in the FNVs in pregnant women with late-onset and early-onset FGR. Data are presented in the format Me (Q1, Q3); *: significance level *p* ≤ 0.05, **: significance level *p* ≤ 0.01, ***: significance level *p* ≤ 0.001, when compared with CG.

We have previously studied the changes in the expression of the same panel of proteins in early-onset FGR [23]; therefore, we decided to conduct a comparative analysis of the protein levels in early-onset and late-onset FGR (Figure 3). This analysis demonstrated the differences in the expression of a number of the studied proteins in the early-onset and late-onset FGR.

In particular, neurotrophin NGF level (0.23, −0.66; *p* ≤ 0.04) was significantly lower, while the levels of NT-3 (−0.01, −0.55 *p* ≤ 0.04) and NT-4 (0.23, −0.48 *p* ≤ 0.007) were significantly higher in early-onset FGR compared to the late-onset condition. At the same time, the BDNF levels differed insignificantly between early-onset and late-onset FGR (−0.15, −0.24; *p* = 0.08). The synaptic protein SYN1 level (−0.83, 0.44 *p* ≤ 0.001) was significantly lower in early-onset FGR compared to the late-onset form, while SYP level (−0.86, 0.98 *p* ≤ 0.001) was significantly higher in late-onset FGR. In the meantime, the expression of NPTX2 (0.82, −0.7 *p* ≤ 0.04) was increased in early-onset FGR. Expression of the proteins involved in sumoylation, SUMO 1 (−0.65, 0.57; *p* ≤ 0.004) and SUMO 2/3/4 (−0.9, 1.19; *p* ≤ 0.0001), was significantly higher in late-onset FGR compared to early-onset FGR, while the level of the neddylation protein NEDD8 (0.4, −0.42; *p* ≤ 0.02) was higher in the early-onset form. In general, the pattern of expression demonstrates a tendency toward a greater change in early-onset FGR compared to the late-onset condition.

### 2.6. Evaluation of the Association Between FNV Protein Expression and Clinical Parameters in the Pregnant Women with Late-Onset FGR

**Figure 4:**
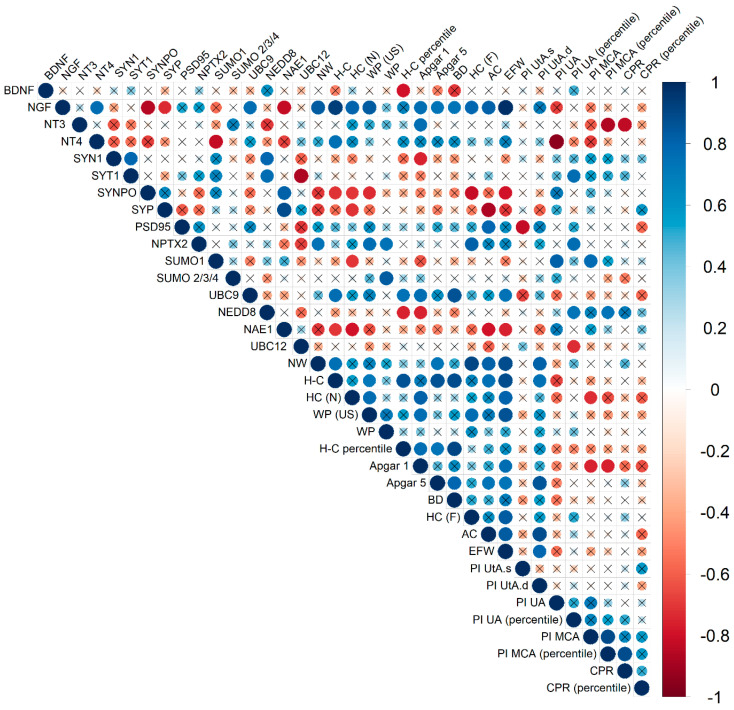
Correlation matrix obtained from the analysis of correlations between protein expression in FNVs and clinical parameters in late-onset FGR. The diameter of the circles and the color indication (according to the scale on the right) are proportional to the correlation coefficient; the fields where the statistical significance of the correlation coefficients is greater than 0.05 are crossed out. SYT1—Synaptotagmin 1, SYN1—Synapsin 1, SYP—Synaptophysin, SYNPO—Synaptopodin, NW—newborn weight, H-C—heel-crown, H-C (percentile)—heel-crown percentile, HC(N)—head circumference of newborn, WP—weight percentile, WP (US)—weight percentile (ultrasound), BD—biparietal diameter of the fetus, HC(F)—head circumference of fetus, AC—abdominal circumference, EFW—estimated fetal weight, PI UtA.s.—Pulsatility index of Uterine Artery (left), PI UtA.d.—Pulsatility index of uterine artery (right), PI UA—Pulsatility index of umbilical artery, PI UA (percentile)—Pulsatility index of umbilical artery percentile, PI MCA—Pulsatility index of middle cerebral artery, PI MCA (percentile)—Pulsatility index of middle cerebral artery percentile, CPR—cerebral placental ratio, CPR (percentile)—cerebral placental ratio percentile.

To assess the association between FNV protein expression and clinical data, we used the nonparametric Spearman’s rank correlation. Some parameters could not be determined in the comparison group according to the examination algorithm (Figure 4).

Correlations with expression patterns of FNV proteins were established for a number of parameters, including both ultrasound biometric markers of the fetus and the clinical features of the newborns. It should be noted that the greatest number of parameters significantly correlated with the pro-NGF level, namely, newborn’s weight (r = 0.83; *p* ≤ 0.01), crown-heel length (r = 0.93; *p* ≤ 0.0009), head circumference (r = 0.8; *p* ≤ 0.01), Apgar score 1 (r = 0.76; *p* ≤ 0.03), Apgar score 5 (r = 0.73; *p* ≤ 0.03), biparietal diameter of the fetus (r = 0.73; *p* ≤ 0.03), head circumference of the fetus (r = 0.79; *p* ≤ 0.02), fetal abdominal circumference (r = 0.81; *p* ≤ 0.02), weight percentile (ultrasound) (r = 0.83; *p* ≤ 0.01) and estimated fetal weight (r = 0.98; *p* ≤ 0.0004). In the meantime, pro-BDNF expression significantly correlated only with crown-heel length (r = 0.79; *p* ≤ 0.02). Associations with biometric parameters and parameters of the newborn were also established for a number of synaptic proteins. SYNPO level negatively correlated with weight percentile (ultrasound) (r = −0.74; *p* ≤ 0.04), fetal head circumference (r = −0.81; *p* ≤ 0.02) and estimated fetal weight (r = −0.79; *p* ≤ 0.02), crown-heel length (r = −0.72; *p* ≤ 0.04) and head circumference of newborn (r = −0.71; *p* ≤ 0.04).

Among the sumoylation and neddylation proteins, significant correlations were found between SUMO 2/3/4 levels and weight percentile based on ultrasound examination (r = 0.83; *p* ≤ 0.01), while NAE1 levels correlated with ultrasound parameters: fetal abdominal circumference (r = −0.79; *p* ≤ 0.02), and estimated fetal weight (r = −0.76; *p* ≤ 0.03) and neonatal parameters: crown-heel length (r = −0.79; *p* ≤ 0.02) and head circumference (r = −0.79; *p* ≤ 0.02). It should be noted that only UBC12 expression correlated with PI UA (percentile) (r = −0.73; *p* ≤ 0.03).

Notably, in addition to the association with clinical parameters, the studied proteins correlated with each other with high coefficients. An association was established between the neurotrophin pro-NGF level and the synaptic proteins SYNPO (r = −0.86; *p* ≤ 0.01) and SYP (r = −0.74; *p* ≤ 0.04), as well as the neddylation protein NAE1 (r = −0.83; *p* ≤ 0.01). Notably, the latter also correlated with SYNPO (r = 0.81; *p* ≤ 0.02) and SYP (r = 0.88; *p* ≤ 0.007).

As noted above, no statistically significant difference in expression was found for a number of FNV proteins between the comparison group and the FGR group. However, significant correlations were found for some of them. In particular, NPTX2 level correlated with weight parameters, namely, newborn weight (r = 0.74; *p* ≤ 0.04), ultrasound weight percentile (r = 0.76; *p* ≤ 0.03), and neonatal weight percentile (r = 0.74; *p* ≤ 0.04). In addition, its level also correlated with fetal head circumference (r = 0.81; *p* ≤ 0.02) and PI UA (percentile) (r = 0.76; *p* ≤ 0.02). Significant correlations were found for the levels of sumoylation proteins SUMO 1 and UBC9. In particular, SUMO 1 expression negatively correlated with the head circumference of the newborn (r = −0.71; *p* ≤ 0.04), and positively correlated with PI UA (r = 0.81; *p* ≤ 0.02) and PI MCA (r = 0.77; *p* ≤ 0.02). The UBC9 level was associated with neonatal crown-heel length (r = 0.76; *p* ≤ 0.02), Apgar 1 (r = 0.76; *p* ≤ 0.03), biparietal diameter of fetus (r = 0.86; *p* ≤ 0.005), and estimated fetal weight (r = 0.74; *p* ≤ 0.04). Significant negative correlations were also established for the neddylation protein NEDD8 with neonatal crown-heel length (percentile) (r = −0.76; *p* ≤ 0.03) and Apgar 1 (r = −0.76; *p* ≤ 0.03). It is interesting to note that the NEDD8 level positively correlated with Doppler parameters of fetal hemodynamics: percentile of PI UA (r = 0.76; *p* ≤ 0.03) and PI MCA (r = 0.76; *p* ≤ 0.03), and CPR (r = 0.7; *p* ≤ 0.05).

Taking into account the above-presented (Section 2.5) comparative analysis of the expression levels of the studied FNVs proteins in early-onset and late-onset FGR, we analyzed the data in the context of the established associations (Table 3).

It is important to emphasize that the levels of the studied proteins correlated with a larger number of parameters in late-onset FGR than in early-onset FGR. Differences in protein expression patterns were also observed between the FGR forms. In particular, among the neurotrophins, the association with pro-BDNF was more pronounced in early-onset FGR, while pro-NGF levels were associated with prenatal and postnatal parameters of weight, length, and the Apgar score in late-onset FGR. Furthermore, correlations with synaptic proteins, SYP and SYNPO, were observed in late-onset FGR. It is also interesting to note the differences in correlations with sumoylation proteins SUMO2/3/4, in late-onset FGR, and UBC9 in the early-onset form. As for neddylation proteins, a similar pattern was observed for the levels of the activating enzyme NAE1 in late-onset FGR, while correlations with UBC12 levels were observed in both early-onset and late-onset FGR. Moreover, the expression of only two conjugating enzymes, UBC9 (sumoylation) and UBC12 (neddylation), correlated with PI UtA and PI UA.

### 2.7. Association of Changes in FNVs Protein Expression with Neonatal Morbidity in Late-Onset FGR

**Figure 5:**
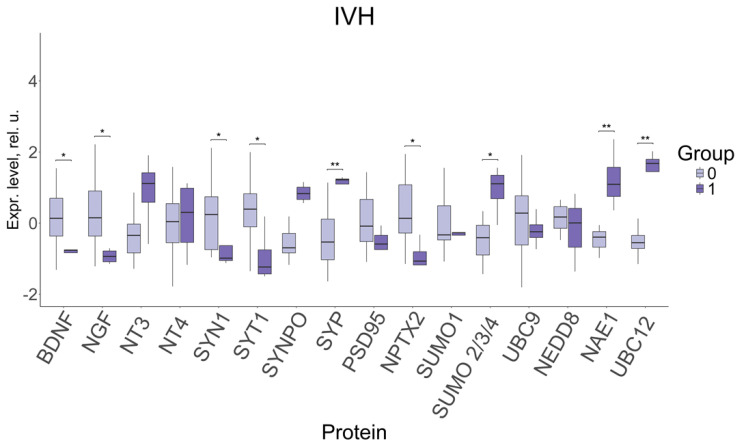
Changes in FNV protein expression in newborns with IVH, depending on the presence of the disorder (1) or its absence (0). Data is presented in the format Me (Q1, Q3); SYT1—Synaptotagmin 1, SYN1—Synapsin 1, SYP—Synaptophysin, SYNPO—Synaptopodin. *: significance level *p* ≤ 0.05, **: significance level *p* ≤ 0.01 when compared with CG.

The changes in the expression levels of FNV proteins were evaluated depending on the occurrence of neonatal complications in late-onset FGR (Figure 5).

A significant decrease in the levels of neurotrophins BDNF (−0.76, 0.14; *p* ≤ 0.01) and NGF (−0.94, 0.15; *p* ≤ 0.01); of the proteins involved in the modulation of synaptic transmission and the release of neurotransmitters: SYN1 (−0.99, 0.24; *p* ≤ 0.04) and SYT1 (−1.24, 0.39; *p* ≤ 0.01); and of NPTX2 protein (−1.07, 0.14; *p* ≤ 0.01), involved in the formation of excitatory synapses and clustering of glutamate receptors, was found to be associated with IVH in neonates with late-onset FGR. Moreover, the levels of SYP synaptic protein (1.22, −0.53; *p* ≤ 0.002); sumoylation proteins SUMO 2/3/4 (1.11, −0.41; *p* ≤ 0.04); and neddylation proteins NAE1 (1.09, −0.4; *p* ≤ 0.004) and UBC12 (1.68, −0.55; *p* ≤ 0.001), demonstrated a significant increase in IVH.

It should be noted that, among the neonatal complications associated with impaired cerebral blood flow in fetuses with late-onset growth restriction, significant differences with the comparison group were found for IVH and subependymal cysts (see Table 2).

## 3. Discussion

The possibilities for identifying prenatal brain damage, which could be considered as an early marker of fetal growth restriction, allowing for the prediction of short-term and long-term adverse effects on the development of the nervous system, are not yet employed. Neuronal dysfunction of the fetal brain due to placental insufficiency can occur before cerebrovascular changes in hemodynamics are found at Doppler ultrasonography [26] and, therefore, is not timely diagnosed. Beyond that, the degree of nervous system dysfunction depends on the timing of the FGR onset (early or late) and the gestational age at the time of birth (preterm or full-term) [27]. Unlike early-onset FGR, the diagnosis of the late-onset condition is difficult due to the low prognostic value of tests used for early stages, with discrepancies regarding the entity of low-birth-weight fetus and growth restriction at birth, as well as changes in Doppler fetoplacental parameters at different stages of pregnancy [28].

Within this paradigm, we used a new approach to identify damage markers, so-called “fetal brain liquid biopsy”. It involved isolating a subpopulation of FNVs using immunoprecipitation from maternal plasma at late-onset FGR, followed by Western blot analysis of the expression patterns of a number of neuronal proteins within the original subpopulation. It should be emphasized that the assessment of the studied neuronal protein levels has the highest specificity, since it rules out the possibility of contamination from other sources.

According to the stages of development of the fetal nervous system, by the beginning of the 32nd gestational week, the six-layer cortex with a full set of neurons is already fully formed, while the growth of axons, dendrites, and the formation of synapses still continue [10,11]. Endogenous neurotrophic factors in the developing brain at this stage are powerful regulators of neuronal differentiation and the establishment of synaptic connections [29]. The role of neurotrophins is crucial for neurogenesis and brain plasticity [30]. The significant decrease in the expression of key neurotrophins such as BDNF and NGF, which we have observed in late-onset FGR, may be directly related to hypoxia due to placental insufficiency, providing a damaging effect on the grey and white matter of the brain. It should be emphasized that the detected sizes of the studied proteins corresponded to their precursors, pro-BDNF (~30–32 kDa) and pro-NGF (~32–34 kDa). At the same time, we did not detect the mature forms of neurotrophins in FNVs. Neurotrophin precursors are known to perform functions opposite to their mature forms, primarily pro-apoptotic. This feature depends on the type of cellular receptor to which the precursor proteins bind [31]. It was suggested that the conversion of pro-BDNF to BDNF plays a significant role in hippocampal synaptic plasticity [32]. According to Yang F. et al., a deficiency in the conversion of pro-BDNF to its mature form is associated with the development of autism spectrum disorders. Mice with this deficiency exhibited decreased dendritic complexity, a decrease in the number of mature spines, and an increase in the number of immature spines, as well as altered synaptic protein levels. Moreover, excessive pro-BDNF expression combined with a deficiency of mature BDNF can also lead to impaired neurogenesis and synaptic dysfunction [33]. As for NGF precursor, it has been reported that its deficiency leads to a decrease in the expression of the low-affinity receptor p75^NTR^, thereby improving neurogenesis in Alzheimer’s disease [34]. According to other studies, activation of p75^NTR^ pro-NGF receptors through a JNK and SYN phosphorylation-mediated mechanism eliminates the deficit in long-term potentiation in the hippocampus in animal models [35].

Taking into account the reduced proneurotrophin levels in our study, we suggest that their deficiency and the absence of mature forms in neuronal exosomes from growth-restricted fetuses may be due to both impaired conversion of pro-BDNF to BDNF and mechanisms of signal-mediated activation of pro-NGF. Furthermore, the expression of SYN1, a neuronal phosphoprotein involved in the early stages of neuronal development and growth dynamics, regulation of synaptogenesis, and neurotransmitter release in presynaptic terminals through reversible binding of synaptic vesicles to the actin cytoskeleton, was also significantly decreased in the late-onset FGR compared to the uncomplicated pregnancy.

SYN1 deficiency may lead to cognitive impairment [36]. Interestingly, one study found mutations in the SYN1 gene associated with autism spectrum disorders and epilepsy [37]. Deletions in this gene are associated with decreased synaptic vesicular complexes at nerve terminals, and their limited availability for release [38]. A research by Qi C-C. et al. showed that imbalances in BDNF expression in the hippocampus or prefrontal cortex were associated with changes in synaptic plasticity, including reduced expression of SYN1, SYT1, and PSD95 in patients with Alzheimer’s disease [39,40]. It is also known that BDNF can act as a presynaptic activator of the H-Ras/Erk/SYN1 signaling pathway, increasing the release of glutamate from synaptosomes through Erk-dependent phosphorylation of SYN1 [38,41]. Considering the close functional connections of neurotrophins and synaptic proteins, we suggest that the decrease in SYN1 expression may also be due to a deficiency in pro-neurotrophin levels.

It should be noted that our research found increased expression of another synaptic vesicle protein (SYP) in FNVs in late-onset FGR, particularly, its conjugated fragment ~57–61 kDa in size. Unlike the main peripheral proteins, synapsins, SYP is the main integral membrane protein of synaptic vesicles [42]. Its expression reflects the processes of neuronal maturation and the formation of neural connections. Some studies suggested that SYP is necessary for maintaining communication in highly active circuits to ensure stable transfer of the vesicle-associated membrane protein synaptobrevin 2 (VAMP2, part of the SNARE complex) and its targeting at synaptic vesicles for the subsequent release of neurotransmitters [43,44,45]. Based on this protein function, we assume that the SYP conjugated form possibly indicates its bonding with VAMP2. However, this hypothesis requires further reliable verification, including assessment of cross-validation protein expression using Western blotting.

Unexpectedly for us, an elevated SYNPO level in the FNVs was found in late-onset FGR. This actin-binding protein is a key mediator of synaptic plasticity in dendritic spines during long-term potentiation in the CA1 region of the hippocampus and glutamate-induced Ca^2+^ release [46,47]. Reduced SYNPO expression resulted in long-term potentiation deficiency and impaired spatial learning in animal models [48]. However, according to Korkotyan E. et al., a transient increase of [Ca^2+^] caused by glutamate can lead to an augmentation and formation of new dendritic spines containing SYNPO, i.e., an increase in plasticity [46]. The study by Vlachos A et al. indicates that decreased neuronal activity leads to a decrease in the intracellular [Ca^2+^] pool, thereby promoting compensatory SYNPO cluster size, causing, in turn, an accumulation of AMPA receptors in excitatory postsynapses [49].

Taking into account the data mentioned above, we hypothesize that increased SYNPO expression in the FNVs in fetuses with late-onset growth restriction may be due to the activation of AMPA receptors, which leads to an increase in [Ca^2+^] influx and, consequently, clusters containing SYNPO, as a compensatory response to decreased neurotrophin levels. Furthermore, the trend toward decreased NPTX2 expression identified in our study may also indicate a decrease in synaptic plasticity, since NPTX2, by binding directly to glutamate AMPA receptors, induces postsynaptic excitatory synapses [50]. Interestingly, according to Goetzl L. et al., intrauterine exposure of fetal brain to ethanol suppressed SYNPO expression in fetal neuronal exosomes isolated from maternal plasma. A discrepancy between our data and the results of Goetzl L. et al. most likely can be explained by different etiologies, since fetal alcohol syndrome is characterized by a direct teratogenic effect on the fetus, leading to a disruption of the molecular structure of cells, in contrast to placental dysfunction in late-onset FGR associated with chronic hypoxia.

Though the proteins that perform post-translational modifications, i.e., sumoylation and neddylation, were chosen for a certain scope of research, their functions are closely related to the overall development of the nervous system [24,25]. These modifications regulate fundamental processes in the brain, including neuron maturation, synapse formation and plasticity, and neurotransmission. An imbalance in their regulation is believed to contribute to the development of various neurological disorders [51]. It should be noted that sumoylation and neddylation are performed by the specific protein enzymes: SUMO 1-4 and UBC9 (sumoylation), and NEDD8, NAE1 (APPBP1), and UBC12 (neddylation). Since both processes are reversible, appropriate deconjugating enzymes are required: SENP 1-3 and SENP 5-7 to reverse sumoylation [52], and SENP 8 and USP21 to reverse neddylation [53].

We found a significant increase in SUMO 2/3/4 (particularly, its conjugated form with size ~55–60 kDa) expression in late-onset FGR, compared to noncomplicated pregnancy. At the same time, the expression of the SUMO1 and UBC9 conjugated forms was insignificant, in contrast to early-onset FGR, where this expression was increased [23]. It is important to note that the sequences of SUMO1 and SUMO 2/3 differ by approximately 50% [54]; this explains why the differences in conjugation dynamics and responses to cellular stress were observed [54,55]. Very low levels of SUMO2/3 conjugated proteins were detected in the undamaged brain. However, after transient cerebral ischemia and during deep hypothermia, they were significantly higher [56,57,58,59]. Importantly, inhibition of sumoylation resulted in a decrease in the number of surface AMPA receptors during the induction of long-term potentiation at synapses, limiting the function of the latter [60]. Various stimuli trigger sumoylation of certain presynaptic proteins, in order to either suppress or stimulate neurotransmitter release [61]. Strong evidence has been provided to support the concept that elevated levels of sumoylated proteins may promote tolerance to ischemic stress and provide neuroprotection during ischemic preconditioning [62]. In this context, we hypothesize that changes in the levels of conjugated SUMO2/3/4 fragments indicate a process of active sumoylation of certain protein substrates in fetal neurons, including synaptic ones, which may be part of both a compensatory endogenous response aimed at maintaining neuronal function and a response to the stress caused by placental insufficiency in FGR.

Interestingly, levels of the neddylation-activating enzyme NAE 1 and the conjugating enzyme UBC 12 were also elevated in late-onset FGR. Similar to sumoylation, the detected proteins may probably indicate neddylation of certain substrates. Inhibition of the neddylation pathway is known to lead to disruption of the cytoskeleton, neurite outgrowth, and dendritic spine destabilization, affecting synaptic transmission and plasticity, probably through the impact on protein substrates important for the latter functions [63,64]. Despite the lack of statistical significance, we observed a trend toward an increase in NEDD8 level. Intriguingly, in our previous study, its level was significantly increased in early-onset FGR, whereas NAE1 levels were reduced, without reaching the significance threshold. Post-translational modifications influence neuronal development to varying degrees. As shown previously, the correct balance between various post-translational protein modifications, rather than substrate expression levels, is crucial for further development of the axonal and dendritic network [65]. However, the comparative analysis revealed that changes in the expression of neurotrophins and synaptic proteins were more pronounced in the early-onset FGR compared to the late-onset disorder, which may indicate the activation of various signaling pathways initiated by post-translational modifications. Furthermore, we propose that the differences in the expression levels of the proteins of sumoylation and neddylation may be distinctive features of the early-onset and late-onset FGR pathogenesis. It should be emphasized that our hypothesis concerning the conjugated forms of sumoylation and neddylation proteins requires further reliable verification.

A cerebral hemodynamic response occurs only with progressive hypoxia [66]. The fetus adapts to hypoxemia by a decrease in tissue oxygen consumption. Fetuses with late-onset growth restriction, unlike those with early-onset conditions, may initially exhibit an acute hypoxic response followed by chronic hypoxia [67]. The increased blood flow in the brain under hypoxic conditions, which occurs during cerebral redistribution, may lead to oxidative stress in tissues already affected by hypoxia/ischemia. In this regard, some researchers consider that antenatal adaptation (i.e., a “brain sparing effect”), which is observed in late-onset FGR, does not really protect the fetal brain but, instead, can be considered as an early predictor of unfavorable neurological outcomes. The increased levels of post-translational proteins in our study, reflecting tolerance to ischemic stress, may be considered as a presumptive assumption in favor of this statement.

It is important to note that the identified changes in the FNV protein expression pattern in late-onset FGR correlated with a range of clinical parameters, including ultrasound biometric markers of the fetus (head circumference, abdominal circumference, biparietal diameter, and estimated fetal weight) and neonatal state assessments (Apgar scores 1 and 5, and birth weight). Interestingly, only UBC12 levels correlated with PI UA (percentile) in late-onset FGR, while UBC9 levels correlated with PI UtA.d in early-onset FGR. Furthermore, no significant correlations were found with PI MCA, which is a criterion for assessing fetal hemodynamics in late-onset growth restriction. Since blood flow in the periventricular zones of the fetal brain directly depends on systemic hemodynamics, its disturbances can be caused by both hypoperfusion and ischemia of the gray and white matter of the brain, as well as by IVH. According to the analysis of the structure of neonatal morbidity associated with cerebrovascular accidents in fetuses with late-onset growth restriction, significant differences were found for IVH and subependymal cysts (Table 2). It is difficult to objectively assess the degree of periventricular ischemia in the first 48 h after birth using the routinely available tools. Our study found that IVH was associated with a significant decrease in the levels of neurotrophin proteins (pro-BDNF, pro-NGF); the levels of proteins involved in the modulation of synaptic transmission and neurotransmitter release (SYN1, SYT1, NPTX2); and a significant increase in the levels of SYP, sumoylation proteins (SUMO 2/3/4), and neddylation proteins (NAE1, UBC12), in neonates with late-onset growth restriction.

## 4. Materials and Methods

### 4.1. Design of the Study and Cohorts of Pregnant Women

**Figure 6:**
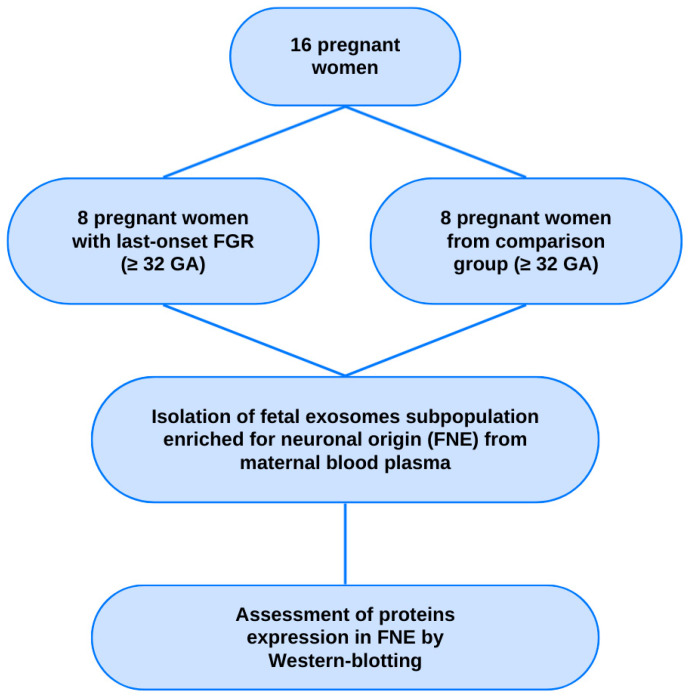
Flowchart of the study population. GA—gestational age.

The study included pregnant women under maternal care at the Federal State Budget Institution “National Medical Research Center for Obstetrics, Gynecology and Perinatology named after Academician V.I. Kulakov” of the Ministry of Health of the Russian Federation. Pregnant patients with late-onset FGR (*n* = 8; ≥32 weeks of gestation) using the Delphi procedure were selected for the pilot study [12]. The comparison group comprised pregnant women at similar terms of gestation (*n* = 8; ≥32 weeks of pregnancy) with uncomplicated pregnancy and no signs of any disorders in the fetuses according to the routine antenatal tests (Figure 6).

The late-onset FGR diagnosis was based on the following absolute criteria (the estimated fetal body weight below the 3rd percentile for gestational age or the abdominal circumference less than the 3rd percentile), and relative criteria, at least two of the following: 1. Fetal abdominal circumference and/or estimated fetal weight less than 10th percentile; 2. Growth dynamics of abdominal circumference and/or estimated fetal weight, crossing more than two quartiles on the percentile growth charts; 3. Cerebro-placental ratio less than the 5th percentile, or PI (pulsatility index) in the umbilical arteries over the 95th percentile. In addition, amniotic fluid scarcity (oligohydramnion and anhydramnion) in pregnant women was determined using the following ultrasonographic diagnostic criteria: single deepest vertical pocket (SDVP) < 2 cm, and amniotic fluid index (AFI) ≤ 5 cm.

Doppler ultrasound (Voluson E10, GE Healthcare Technologies, Milwaukee, WI, USA) was used for evaluation of the velocity curves of blood flow (PI) in the uterine, middle cerebral, and umbilical arteries of the fetus. The informed consent of patients included in the study was carried out in accordance with the Helsinki Declaration and the Approval by the Commission of Biomedical Ethics (#10, 20 October 2022) at the “National Medical Research Center for Obstetrics, Gynecology and Perinatology named after Academician V.I. Kulakov” of the Ministry of Health of the Russian Federation.

The neonates were assessed in accordance with common criteria, which included birth weight and Apgar score. The body length–weight values are presented in accordance with INTERGROWTH-21st and the Fenton growth chart.

Comparative analysis of FNVs protein expression in early- and late-onset FGR was carried out between the data obtained in the current study for late-onset FGR, and the data obtained in our previous study for early-onset FGR and published results [23].

### 4.2. Isolation of Fetal Vesicles Subpopulation Enriched for Neuronal Origin (FNVs) from the Plasma Blood Samples of Pregnant Women

Isolation of total maternal vesicles by ExoQuick Plasma Prep and Exosome Precipitation kit (Cat.# EXOQ5TM-1, System Biosciences, Palo Alto, CA, USA) was carried out from plasma blood samples of study patients according to the manufacturer’s instructions. We used 250 µL of total maternal vesicles for FNVs subpopulation isolation according to protocols described by Goetzl L. et al. [21,22]. Preliminarily, the blood samples were collected into VACUETTE^®^ tubes EDTA (Becton Dickinson, Mississauga, ON, Canada). Subsequently, the samples were centrifuged at 300× *g*, 4 °C for 20 min. The supernatant was collected and centrifuged at 16,000× *g* for 10 min. The finished supernatant was transferred into a new tube for subsequent vesicle isolation. Contactin-2/TAG1 (CNTN2) is an axonal surface glycoprotein that is highly expressed by the fetus during pregnancy; it was used to immunoprecipitate FNVs [68]. Briefly, 2 μg mouse monoclonal antibody Contactin-2/TAG1 (Cat.# MAB17141 clone 372913, R&D Systems, Fisher Scientific, Waltham, MA, USA) preliminary biotinylated (Cat.# 21217 EZ-Link™ Sulfo-NHS-Biotin, Thermo Fisher Scientific, Waltham, MA, USA) was diluted in 50 μL of 3% bovine serum albumin (BSA) (Cat.# 37525 Blocker™ BSA (10×) in PBS, Thermo Fisher Scientific, Waltham, MA, USA). Further, incubation with maternal vesicles was performed for 1.5 h at 20 °C, and mixed gently by inversion. The next incubation immunoprecipitated antibodies with streptavidin (Cat.# 53116 Pierce™ Streptavidin Plus UltraLink™ Resin, Thermo Fisher Scientific, Waltham, MA, USA) in 50 µL 3% BSA and was carried out for an hour at 20 °C, mixed gently by inversion. The obtained mixture was centrifuged for 10 min at 300× *g*, 4 °C. The supernatant was removed, and the pellet was resuspended in 100 μL of cooled 0.1 M glycine (pH 2.5). Futhermore, the resuspended pellet was centrifuge for 10 min at 4000× *g*, 4 °C, After that the supernatant was collected, supplemented with 5 μL of 1 M Tris (pH 8) and 20 μL of 3% BSA, and then mixed with 250 μL of M-PER (Cat.# 78501 M-PER™ Mammalian Protein Extraction Reagent, Thermo Fisher Scientific, Waltham, MA, USA) containing phosphatase and protease inhibitors at a concentration 3 times higher than recommended (Cat.# 78428 Halt™ Phosphatase Inhibitor Single-Use Cocktail, Thermo Fisher Scientific, Waltham, MA, USA; Cat.# 11697498001 cOmplete™ Protease Inhibitor Cocktail, Sigma-Aldrich, Merck, Darmstadt, Germany). The obtained mixture was subjected to freeze/thaw cycles twice. Next, it was aliquoted for subsequent Western blotting. The annotation of the study samples was coded to eliminate study bias.

### 4.3. Western Blotting of FNV Proteins

FNVs proteins were separated in Tris/Tricine/SDS Buffer (12.5%). The semi-dry blotting (Trans-Blot SD™, Cat.# 170-3957, Bio-Rad, Hercules, CA, USA) in a discontinuous buffer system was used for FNVs samples transfer to nitrocellulose membrane (0.45 µm, Cat.# 1620115 Bio-Rad, Hercules, CA, USA). This system included two buffers: 60 mM Tris, 40 mM CAPS, pH 9.6, plus 15% ethanol in the filter paper on the anode side and the same buffers plus 0.1% SDS on the cathode side. The membranes were blocked with 5% NFDM/TBST for 2 h. Furthermore, incubation during an hour at the room temperature with primary antibodies, including BDNF (1:1000, ab205067, Abcam, Waltham, MA, USA), NGF (1:1000, SI79-01 (rabbit), Thermo Fisher Scientific, Waltham, MA, USA), NT3 (1:200, sc-518099, Santa Cruz Biotechnology, Santa Cruz, CA, USA), NT4 (1:200, sc-365444, Santa Cruz Biotechnology, Santa Cruz, CA, USA), SYNAPTOTAGMIN 1 (SYT1) (1:200, sc-136480, Santa Cruz Biotechnology, Santa Cruz, CA, USA), SYNAPSIN 1 (SYN1) (1:1000, ab254349 (rabbit), Abcam, Waltham, MA, USA), SYNAPTOPHYSIN (SYP) (1:1000, sc-17750, Santa Cruz Biotechnology, Santa Cruz, CA, USA), SYNAPTOPODIN (SYNPO) (1:100, sc-515842, Santa Cruz Biotechnology, Santa Cruz, CA, USA), PSD95 (1:200, sc-32291, Santa Cruz Biotechnology, Santa Cruz, CA, USA), Neuronal Pentraxin 2 (NPTX2) (1:1000, ab277523 (rabbit), Abcam, Waltham, MA, USA), NEDD8 (1:1000, ab81264, (rabbit), Abcam, Waltham, MA, USA), NAE1 (APPBP1) (1:100, sc-390002, Santa Cruz Biotechnology, Santa Cruz, CA, USA), UBC12 (1:100, sc-390064, Santa Cruz Biotechnology, Santa Cruz, CA, USA), SUMO 1 (1:200; sc-5308, Santa Cruz Biotechnology, Santa Cruz, CA, USA), SUMO 2/3/4 (1:200, sc-393144, Santa Cruz Biotechnology, Santa Cruz, CA, USA), UBC9 (1:100; sc-271057, Santa Cruz Biotechnology, Santa Cruz, CA, USA), Contactin-2/TAG1 (1:1000; MAB17141, Fisher Scientific, Waltham, MA, USA). Exosomal marker CD81 was used to assess plasma FNVs levels (1:100, sc-166029, Santa Cruz Biotechnology, Santa Cruz, CA, USA). Secondary HRP-conjugated antibodies were incubated for an hour (room temperature) in 1% NFDM/TBST (goat anti-mouse IgG-HRP (ab205719, Abcam, Waltham, MA, USA), goat anti-rabbit IgG-HRP (ab97051, Abcam, Waltham, MA, USA), sc-516102, sc-525408, sc-525409, sc-533670 (Santa Cruz Biotechnology, Santa Cruz, CA, USA)). A SuperSignal West Femto Maximum Sensitivity Substrate Kit (Cat.# 34096, Thermo Scientific, Waltham, MA, USA) was used as a detection reagent. Densitometry was performed using Bio-Rad ImageLab 6.0 software.

### 4.4. Data Analysis

The statistical significance of the difference between the clinical parameters and the proteins expression in the study groups was assessed by the Wilcoxon–Mann–Whitney test using scripts written in the R language v.4.5.2 (https://www.r-project.org/, accessed on 6 January 2026)). The Spearman nonparametric rank correlation method was used to evaluate the relationship between the protein expression and clinical parameters of pregnant women and newborns. The expression of each FNV protein was normalized to the sum of signals from all analyzed proteins in the sample and then standardized. The STRING v.12 database (https://string-db.org/, accessed on 6 January 2026) was used to select the proteins to be studied.

## 5. Conclusions

The isolation of a subpopulation of fetal neuronal vesicles from maternal blood by immunoprecipitation revealed significant changes in the expression patterns of fetal neuronal proteins; these changes may be considered potential markers of fetal brain damage in late-onset FGR. Furthermore, differences in the expression of conjugated proteins of sumoylation and neddylation may be distinctive features of the early- and late-onset FGR.

The main limitation of the study was the sample size; therefore, this work should be considered as a pilot study. Furthermore, the diagnostic potential of the neuronal proteins should be assessed in a large cohort of patients with FGR. Further studies will help determine whether these neuronal proteins can be considered as potential targets for antenatal neuroprotective interventions. At the same time, longitudinal correlation of specific FNV protein patterns with detailed neurodevelopmental assessments at 1–2 years of age will help clarify their true prognostic value.

Overall, the introduction of a “fetal brain liquid biopsy” as a new approach opens up promising prospects for the development of algorithms for early FGR diagnostics based on assessment of the risk of neuronal dysfunction and neurological complications.

## Figures and Tables

**Table 1:** Ultrasound and Doppler data of the study patients.

	≥32 GA		
	Late-Onset FGR Group (*n* = 8)	Comparison Group (*n* = 8)	*p*-Value
Gestational age at time of sample collection, weeks	36.6 (36.0; 38.1)	36.6 (35.5; 38.0)	0.637
Maternal age, years	32 (26.32; 37.43)	35 (29.88; 39.12)	0.405
Preeclampsia, *n* (%)	2 (25)	0 (0)	0.467
Pulsatility index of Uterine Artery (left) (PI UtA.s.)	1.12 (0.77; 1.47)	0.78 (0.64; 0.92)	0.054
Pulsatility index of Uterine Artery (right) (PI UtA.d.)	0.82 (0.65; 0.99)	0.79 (0.70; 0.89)	0.733
Pulsatility index of Umbilical Artery (PI UA)	1.33 (1.12; 1.54)	0.79 (0.72; 0.86)	<0.001
PI UA, percentile	100.0 (99.8; 100.0)	27.0 (18.8; 36.3)	<0.001
Pulsatility index of Middle Cerebral Artery (PI MCA)	1.31 (0.98; 1.64)	1.47 (1.21; 1.73)	0.368
PI MCA, percentile	3 (2; 17)	11 (4; 41)	0.491
Cerebral Placental Ratio (CPR)	1.0 (0.8; 1.3)	1.9 (1.6; 2.1)	<0.001
Cerebral Placental Ratio (CPR), percentile	1 (1; 1)	36 (26; 63)	<0.001
Biparietal diameter (BD), mm	84.9 (81.4; 88.6)	89.1 (85.7; 92.5)	0.072
Head circumference (HC), mm	308.9 (301.2; 316.4)	314.6 (311.9; 331.0)	0.189
Abdominal circumference (AC), mm	286.2 (269.3; 290.1)	301.8 (284.0; 305.7)	0.093
Estimated Fetal Weight (EFW), kg	2.07 (1.88; 2.25)	2.61 (2.31; 2.88)	0.074
EFW, percentile	2.25 (0.82; 3.68)	37.71 (17.11; 58.31)	0.005
Impaired fetoplacental blood flow, *n* (%)	8 (100)	0 (0)	<0.001
Impaired uteroplacental blood flow (left), *n* (%)	4 (50)	1 (12.5)	0.282
Impaired uteroplacental blood flow (right), *n* (%)	2 (25)	0 (0)	0.467
Null end-diastolic blood flow or absence of blood flow in umbilical artery, *n* (%)	0 (0)	0 (0)	N/A
Oligohydramnios, *n* (%)	3 (37.5)	2 (25)	1

N/A—not analyzed. GA—gestational age (weeks). The data are presented in the format Me (Q1; Q3), where Me is the median, and Q1 and Q3 are quartiles.

**Table 2:** Assessment of newborns and neonatal outcomes.

	≥32 GA		
	Newborns with FGR (*n* = 8)	Comparison Group (*n* = 8)	*p*-Value
Birth weight, kg	2.11 (1.88; 2.34)	2.72 (2.60; 2.85)	<0.001
Birth weight, percentile	3.9 (1.74; 6.07)	46.2 (29.5; 62.9)	<0.001
VLBW, *n* (%)	8 (100)	0 (0)	<0.001
APGAR 1 min	8 (7; 8)	8 (8; 8)	0.14
APGAR 5 min	8 (8; 9)	9 (9; 9)	0.04
**Neonatal outcomes:**			
Antenatal pneumonia, *n* (%)	2 (25)	0 (0)	0.46
RDS, *n* (%)	0 (0)	1 (12.5)	1
Pulmonary hypertension, *n* (%)	0 (0)	0 (0)	N/A
Hyperbilirubinemia of prematurity, *n* (%)	2 (25)	3 (37.5)	1
Neonatal asphyxia, *n* (%)	0 (0)	0 (0)	N/A
Cerebral ischemia, *n* (%)	0 (0)	0 (0)	N/A
IVH, *n* (%)	5 (62.5)	0 (0)	0.02
Subependymal cyst, *n* (%)	5 (62.5)	0 (0)	0.02
Increased echogenicity in the PV zones, *n* (%)	1 (12.5)	0 (0)	1
CNS depression, *n* (%)	1 (12.5)	1 (12.5)	1
Signs of vasodilation, increased venous outflow velocity, *n* (%)	0 (0)	0 (0)	N/A
Disseminated intravascular coagulation, *n* (%)	0 (0)	0 (0)	N/A
NEC, *n* (%)	0 (0)	0 (0)	N/A
Muscular dystonia syndrome, *n* (%)	0 (0)	0 (0)	N/A
Hyperkinesis, *n* (%)	0 (0)	0 (0)	N/A
Retinopathy, *n* (%)	2 (25)	0 (0)	0.46
Enlargement of the right and left heart chambers, *n* (%)	3 (37.5)	0 (0)	0.2
Hospital stay, days	13 (5; 19)	5 (5; 8)	0.41
Duration of respiratory support, hours	0 (0; 4)	0 (0; 0)	0.37

VLBW is a very low birth weight. RDS is respiratory distress syndrome. IVH is intraventricular hemorrhage. NEC is necrotizing enterocolitis. N/A—not analysed. GA—gestational age (weeks). The data are presented in the format Me (Q1; Q3), where Me is the median, and Q1 and Q3 are quartiles.

**Table 3:** Comparative analysis of correlation associations in early-onset and late-onset FGR.

Name	Pro-BDNF (r)	Pro-NGF (r)	SYNPO (r)	SYP (r)	SUMO 2/3/4 (r)	UBC9 (r)	NAE1 (r)	UBC12 (r)
	Early-onset FGR							
H-C	−0.81							0.75
H-C percentile	−0.73							0.79
Apgar 1	−0.74							
PI UtA.d						−0.84		
	Late-onset FGR							
NW		0.83						
H-C	−0.79	0.93	−0.72				−0.79	
HC (N)		0.8	−0.71	−0.72			−0.79	
Apgar 1		0.76						
Apgar 5		0.73						
BD		0.73						
HC (F)		0.79	−0.81					
AC		0.81		−0.81				
WP (UZ)		0.83	−0.74		0.83		−0.79	
EFW		0.98	−0.79				−0.76	
PI UA (percentile)								−0.73

The table shows only statistically significant correlations (*p* ≤ 0.05). (r) is the correlation coefficient. The correlation data for early-onset FGR are taken from our previously published article [23]. The colors indicate early-onset and late-onset FGR.

## Data Availability

The original contributions presented in this study are included in the article. Further inquiries can be directed to the corresponding author.

## References

[1] Volochaeva M.V., Timofeeva A.V., Fedorov I.S., Kan N.E., Tyutyunnik V.L., Ryzhova K.O., Gasymova S.R. (2025). A Model for Diagnosing Fetal Growth Restriction Using Functional Diagnostic Methods. Obstet. Gynecol..

[2] Kingdom J., Ashwal E., Lausman A., Liauw J., Soliman N., Figueiro-Filho E., Nash C., Bujold E., Melamed N. (2023). Guideline No. 442: Fetal Growth Restriction: Screening, Diagnosis, and Management in Singleton Pregnancies. J. Obstet. Gynaecol. Can..

[3] Melamed N., Baschat A., Yinon Y., Athanasiadis A., Mecacci F., Figueras F., Berghella V., Nazareth A., Tahlak M., McIntyre H.D. (2021). FIGO (International Federation of Gynecology and Obstetrics) Initiative on Fetal Growth: Best Practice Advice for Screening, Diagnosis, and Management of Fetal Growth Restriction. Int. J. Gynecol. Obstet..

[4] Giouleka S., Tsakiridis I., Mamopoulos A., Kalogiannidis I., Athanasiadis A., Dagklis T. (2023). Fetal Growth Restriction: A Comprehensive Review of Major Guidelines. Obstet. Gynecol. Surv..

[5] Moreno-Fernandez J., Ochoa J.J., Lopez-Frias M., Diaz-Castro J. (2020). Impact of Early Nutrition, Physical Activity and Sleep on the Fetal Programming of Disease in the Pregnancy: A Narrative Review. Nutrients.

[6] Hartkopf J., Schleger F., Keune J., Wiechers C., Pauluschke-Froehlich J., Weiss M., Conzelmann A., Brucker S., Preissl H., Kiefer-Schmidt I. (2018). Impact of Intrauterine Growth Restriction on Cognitive and Motor Development at 2 Years of Age. Front. Physiol..

[7] Kim H.S., Kim E.K., Park H.K., Ahn D.H., Kim M.J., Lee H.J. (2020). Cognitive Outcomes of Children with Very Low Birth Weight at 3 to 5 Years of Age. J. Korean Med. Sci..

[8] Sacchi C., Marino C., Nosarti C., Vieno A., Visentin S., Simonelli A. (2020). Association of Intrauterine Growth Restriction and Small for Gestational Age Status with Childhood Cognitive Outcomes: A Systematic Review and Meta-analysis. JAMA Pediatr..

[9] Leonova A.A., Kan N.E., Tyutyunnik V.L., Serebryakova A.P., Khachaturyan A.A., Pekareva N.A. (2025). Neonatal Complications and Characteristics of Postnatal Development of Infants with Fetal Growth Restriction. Obstet. Gynecol..

[10] Dudink I., Hüppi P.S., Sizonenko S.V., Castillo-Melendez M., Sutherland A.E., Allison B.J., Miller S.L. (2022). Altered Trajectory of Neurodevelopment Associated with Fetal Growth Restriction. Exp. Neurol..

[11] Volpe J.J. (2008). Neurology of the Newborn.

[12] Gordijn S.J., Beune I.M., Thilaganathan B., Papageorghiou A., Baschat A.A., Baker P.N., Silver R.M., Wynia K., Ganzevoort W. (2016). Consensus Definition of Fetal Growth Restriction: A Delphi Procedure. Ultrasound Obstet. Gynecol..

[13] Richter A.E., Salavati S., Kooi E.M.W., den Heijer A.E., Foreman A.B., Schoots M.H., Bilardo C.M., Scherjon S.A., Tanis J.C., Bos A.F. (2020). Fetal Brain-Sparing, Postnatal Cerebral Oxygenation, and Neurodevelopment at 4 Years of Age Following Fetal Growth Restriction. Front. Pediatr..

[14] Polat O.A., Kirlangic M.M., Sahin E., Madendag Y., Evereklioglu C., Horozoglu F., Karaca C. (2024). Role of the Brain-Sparing Effect on Retinopathy of Prematurity in Newborns with Fetal Growth Restriction. Curr. Med. Res. Opin..

[15] Kirlangic M.M., Sahin E., Madendag Y., Vural Yalman M., Akdemir E., Eraslan Sahin M., Col Madendag I., Acmaz G. (2021). The Role of the Brain-Sparing Effect of Growth-Restricted Fetuses in Newborn Germinal Matrix/Intraventricular Hemorrhage. J. Perinat. Med..

[16] Coenen H., Braun J., Köster H., Möllers M., Schmitz R., Steinhard J., Oelmeier K. (2022). Role of Umbilicocerebral and Cerebroplacental Ratios in Prediction of Perinatal Outcome in FGR Pregnancies. Arch. Gynecol. Obstet..

[17] Conde-Agudelo A., Villar J., Kennedy S.H., Papageorghiou A.T. (2018). Predictive Accuracy of Cerebroplacental Ratio for Adverse Perinatal and Neurodevelopmental Outcomes in Suspected Fetal Growth Restriction: Systematic Review and Meta-analysis. Ultrasound Obstet. Gynecol..

[18] Melekoglu R., Yilmaz E., Yasar S., Hatipoglu I., Kahveci B., Sucu M. (2021). The Ability of Various Cerebroplacental Ratio Thresholds to Predict Adverse Neonatal Outcomes in Term Fetuses Exhibiting Late-Onset Fetal Growth Restriction. J. Perinat. Med..

[19] Colombo M., Raposo G., Théry C. (2014). Biogenesis, Secretion, and Intercellular Interactions of Exosomes and Other Extracellular Vesicles. Annu. Rev. Cell Dev. Biol..

[20] Goetzl E.J., Boxer A., Schwartz J.B., Abner E.L., Petersen R.C., Miller B.L., Kapogiannis D. (2015). Altered Lysosomal Proteins in Neural-Derived Plasma Exosomes in Preclinical Alzheimer Disease. Neurology.

[21] Goetzl L., Darbinian N., Goetzl E.J. (2016). Novel Window on Early Human Neurodevelopment via Fetal Exosomes in Maternal Blood. Ann. Clin. Transl. Neurol..

[22] Goetzl L., Darbinian N., Merabova N. (2019). Noninvasive Assessment of Fetal Central Nervous System Insult: Potential Application to Prenatal Diagnosis. Prenat. Diagn..

[23] Gusar V., Kan N., Leonova A., Chagovets V., Tyutyunnik V., Khachatryan Z., Yarotskaya E., Sukhikh G. (2025). Non-Invasive Assessment of Neurogenesis Dysfunction in Fetuses with Early-Onset Growth Restriction Using Fetal Neuronal Exosomes Isolating from Maternal Blood: A Pilot Study. Int. J. Mol. Sci..

[24] Gwizdek C., Cassé F., Martin S. (2013). Protein Sumoylation in Brain Development, Neuronal Morphology and Spinogenesis. Neuromol. Med..

[25] Rabut G., Peter M. (2008). Function and Regulation of Protein Neddylation. EMBO Rep..

[26] Tolsa C.B., Zimine S., Warfield S.K., Freschi M., Sancho Rossignol A., Lazeyras F., Hanquinet S., Pfizenmaier M., Huppi P.S. (2004). Early Alteration of Structural and Functional Brain Development in Premature Infants Born with Intrauterine Growth Restriction. Pediatr. Res..

[27] Miller S.L., Huppi P.S., Mallard C. (2016). The Consequences of Fetal Growth Restriction on Brain Structure and Neurodevelopmental Outcome. J. Physiol..

[28] MacDonald T.M., McCarthy E.A., Walker S.P. (2015). Shining Light in Dark Corners: Diagnosis and Management of Late-Onset Fetal Growth Restriction. Aust. N. Z. J. Obstet. Gynaecol..

[29] Fleiss B., Coleman H.A., Castillo-Melendez M., Ireland Z., Walker D.W., Parkington H.C. (2011). Effects of Birth Asphyxia on Neonatal Hippocampal Structure and Function in the Spiny Mouse. Int. J. Dev. Neurosci..

[30] Sahay A., Kale A., Joshi S. (2020). Role of Neurotrophins in Pregnancy and Offspring Brain Development. Neuropeptides.

[31] Teng H.K., Teng K.K., Lee R., Wright S., Tevar S., Almeida R.D., Kermani P., Torkin R., Chen Z.Y., Lee F.S. (2005). ProBDNF Induces Neuronal Apoptosis via Activation of a Receptor Complex of p75^NTR^ and Sortilin. J. Neurosci..

[32] Pang P.T., Teng H.K., Zaitsev E., Woo N.T., Sakata K., Zhen S., Teng K.K., Yung W.H., Hempstead B.L., Lu B. (2004). Cleavage of proBDNF by tPA/Plasmin Is Essential for Long-Term Hippocampal Plasticity. Science.

[33] Yang F., You H., Mizui T., Ishikawa Y., Takao K., Miyakawa T., Li X., Bai T., Xia K., Zhang L. (2024). Inhibiting proBDNF to Mature BDNF Conversion Leads to ASD-like Phenotypes in Vivo. Mol. Psychiatry.

[34] Olabiyi B.F., Fleitas C., Zammou B., Ferrer I., Rampon C., Egea J., Espinet C. (2021). proNGF Involvement in the Adult Neurogenesis Dysfunction in Alzheimer’s Disease. Int. J. Mol. Sci..

[35] La Rosa L.R., Matrone C., Ferraina C., Panico M.B., Piccirilli S., Di Certo M.G., Strimpakos G., Mercuri N.B., Calissano P., D’Amelio M. (2012). Age-Related Changes of Hippocampal Synaptic Plasticity in AβPP-Null Mice are Restored by NGF Through p75NTR. J. Alzheimers Dis..

[36] Corradi A., Zanardi A., Giacomini C., Onofri F., Valtorta F., Zoli M., Benfenati F. (2008). Synapsin-I- and Synapsin-II-Null Mice Display an Increased Age-Dependent Cognitive Impairment. J. Cell Sci..

[37] Fassio A., Patry L., Congia S., Onofri F., Piton A., Gauthier J., Pozzi D., Messa M., Defranchi E., Fadda M. (2011). SYN1 Loss-of-Function Mutations in Autism and Partial Epilepsy Cause Impaired Synaptic Function. Hum. Mol. Genet..

[38] Cesca F., Baldelli P., Valtorta F., Benfenati F. (2010). The Synapsins: Key Actors of Synapse Function and Plasticity. Prog. Neurobiol..

[39] Liu S., Fan M., Xu J.X., Yang L.J., Qi C.C., Xia Q.R., Ge J.F. (2022). Exosomes Derived from Bone-Marrow Mesenchymal Stem Cells Alleviate Cognitive Decline in AD-like Mice by Improving BDNF-Related Neuropathology. J. Neuroinflamm..

[40] Qi C.C., Chen X.X., Gao X.R., Xu J.X., Liu S., Ge J.F. (2021). Impaired Learning and Memory Ability Induced by a Bilaterally Hippocampal Injection of Streptozotocin in Mice: Involved With the Adaptive Changes of Synaptic Plasticity. Front. Aging Neurosci..

[41] Jovanovic J.N., Czernik A.J., Fienberg A.A., Greengard P., Sihra T.S. (2000). Synapsins as Mediators of BDNF-Enhanced Neurotransmitter Release. Nat. Neurosci..

[42] Thiel G. (1993). Synapsin I, Synapsin II, and Synaptophysin: Marker Proteins of Synaptic Vesicles. Brain Pathol..

[43] Pennuto M., Bonanomi D., Benfenati F., Valtorta F. (2003). Synaptophysin I Controls the Targeting of VAMP2/Synaptobrevin II to Synaptic Vesicles. Mol. Biol. Cell.

[44] White D.N., Stowell M.H.B. (2021). Room for Two: The Synaptophysin/Synaptobrevin Complex. Front. Synaptic Neurosci..

[45] Cousin M.A. (2021). Synaptophysin-Dependent Synaptobrevin-2 Trafficking at the Presynapse-Mechanism and Function. J. Neurochem..

[46] Korkotian E., Frotscher M., Segal M. (2014). Synaptopodin Regulates Spine Plasticity: Mediation by Calcium Stores. J. Neurosci..

[47] Vlachos A., Ikenberg B., Lenz M., Becker D., Reifenberg K., Bas-Orth C., Deller T. (2013). Synaptopodin Regulates Denervation-Induced Homeostatic Synaptic Plasticity. Proc. Natl. Acad. Sci. USA.

[48] Jedlicka P., Deller T. (2017). Understanding the Role of Synaptopodin and the Spine Apparatus in Hebbian Synaptic Plasticity—New Perspectives and the Need for Computational Modeling. Neurobiol. Learn. Mem..

[49] Vlachos A., Korkotian E., Schonfeld E., Copanaki E., Deller T., Segal M. (2009). Synaptopodin Regulates Plasticity of Dendritic Spines in Hippocampal Neurons. J. Neurosci..

[50] Xiao M.F., Xu D., Craig M.T., Pelkey K.A., Chien C.C., Shi Y., Zhang J., Resnick S., Pletnikova O., Salmon D. (2017). NPTX2 and Cognitive Dysfunction in Alzheimer’s Disease. eLife.

[51] Folci A., Mirabella F., Fossati M. (2020). Ubiquitin and Ubiquitin-Like Proteins in the Critical Equilibrium between Synapse Physiology and Intellectual Disability. eNeuro.

[52] Nayak A., Müller S. (2014). SUMO-Specific Proteases/Isopeptidases: SENPs and Beyond. Genome Biol..

[53] He X., Zhu A., Feng J., Wang X. (2022). Role of Neddylation in Neurological Development and Diseases. Biotechnol. Appl. Biochem..

[54] Silveirinha V., Stephens G.J., Cimarosti H. (2013). Molecular Targets Underlying SUMO-Mediated Neuroprotection in Brain Ischemia. J. Neurochem..

[55] Saitoh H., Hinchey J. (2000). Functional Heterogeneity of Small Ubiquitin-Related Protein Modifiers SUMO-1 versus SUMO-2/3. J. Biol. Chem..

[56] Lee Y.J., Miyake S., Wakita H., McMullen D.C., Azuma Y., Auh S., Hallenbeck J.M. (2007). Protein SUMOylation is Massively Increased in Hibernation Torpor and is Critical for the Cytoprotection Provided by Ischemic Preconditioning and Hypothermia in SHSY5Y Cells. J. Cereb. Blood Flow Metab..

[57] Cimarosti H., Henley J.M. (2008). Investigating the Mechanisms Underlying Neuronal Death in Ischemia Using In Vitro Oxygen-Glucose Deprivation: Potential Involvement of Protein SUMOylation. Neuroscientist.

[58] Yang W., Sheng H., Warner D.S., Paschen W. (2008). Transient Focal Cerebral Ischemia Induces a Dramatic Activation of Small Ubiquitin-Like Modifier Conjugation. J. Cereb. Blood Flow Metab..

[59] Wang L., Ma Q., Yang W., Mackensen G.B., Paschen W. (2012). Moderate Hypothermia Induces Marked Increase in Levels and Nuclear Accumulation of SUMO2/3-Conjugated Proteins in Neurons. J. Neurochem..

[60] Jaafari N., Konopacki F.A., Owen T.F., Kantamneni S., Rubin P., Craig T.J., Wilkinson K.A., Henley J.M. (2013). SUMOylation Is Required for Glycine-Induced Increases in AMPA Receptor Surface Expression (ChemLTP) in Hippocampal Neurons. PLoS ONE.

[61] Feligioni M., Nishimune A., Henley J.M. (2009). Protein SUMOylation Modulates Calcium Influx and Glutamate Release from Presynaptic Terminals. Eur. J. Neurosci..

[62] Cuomo O., Casamassa A., Brancaccio P., Laudati G., Valsecchi V., Anzilotti S., Vinciguerra A., Pignataro G., Annunziato L. (2020). Sumoylation of Sodium/Calcium Exchanger in Brain Ischemia and Ischemic Preconditioning. Cell Calcium.

[63] Brockmann M.M., Döngi M., Einsfelder U., Körber N., Refojo D., Stein V. (2019). Neddylation Regulates Excitatory Synaptic Transmission and Plasticity. Sci. Rep..

[64] Vogl A.M., Brockmann M.M., Giusti S.A., Maccarrone G., Vercelli C.A., Bauder C.A., Richter J.S., Roselli F., Hafner A.S., Dedic N. (2015). Neddylation Inhibition Impairs Spine Development, Destabilizes Synapses and Deteriorates Cognition. Nat. Neurosci..

[65] Fornasiero E.F., Bonanomi D., Benfenati F., Valtorta F. (2010). The Role of Synapsins in Neuronal Development. Cell. Mol. Life Sci..

[66] Hernandez-Andrade E., Serralde J.A., Cruz-Martinez R. (2012). Can Anomalies of Fetal Brain Circulation be Useful in the Management of Growth Restricted Fetuses?. Prenat. Diagn..

[67] Alves de Alencar Rocha A.K., Allison B.J., Yawno T., Polglase G.R., Sutherland A.E., Malhotra A., Jenkin G., Castillo-Melendez M., Miller S.L. (2017). Early- versus Late-Onset Fetal Growth Restriction Differentially Affects the Development of the Fetal Sheep Brain. Dev. Neurosci..

[68] Masuda T. (2017). Contactin-2/TAG-1, Active on the Front Line for Three Decades. Cell Adhes. Migr..

